# Effect of marker-free transgenic *Chlamydomonas* on the control of *Aedes* mosquito population and on plankton

**DOI:** 10.1186/s13071-022-05647-3

**Published:** 2023-01-18

**Authors:** Xiaowen Fei, Xiaodan Huang, Zhijie Li, Xinghan Li, Changhao He, Sha Xiao, Yajun Li, Xiuxia Zhang, Xiaodong Deng

**Affiliations:** 1 Institute of Tropical Bioscience and Biotechnology, Chinese Academy of Tropical Agricultural Science and Key Laboratory of Biology and Genetic Resources of Tropical Crops of Hainan Province, Hainan Institute for Tropical Agricultural Resources, Haikou, China; 2 Department of Biochemistry and Molecular Biology, Hainan Medical University, Haikou, China; 3 Hainan Provincial Key Laboratory for Functional Components Research and Utilization of Marine Bio-Resources, Haikou, China; 4 Zhanjiang Experimental Station, CATAS, Zhanjiang, China

**Keywords:** Marker-free RNA interference, *Aedes albopictus*, *Chlamydomonas*, High-throughput sequencing, Plankton

## Abstract

**Background:**

More than half of the world’s population suffers from epidemic diseases that are spread by mosquitoes. The primary strategy used to stop the spread of mosquito-borne diseases is vector control. Interference RNA (RNAi) is a powerful tool for controlling insect populations and may be less susceptible to insect resistance than other strategies. However, public concerns have been raised because of the transfer of antibiotic resistance marker genes to environmental microorganisms after integration into the recipient genome, thus allowing the pathogen to acquire resistance. Therefore, in the present study, we modified the 3-hydroxykynurenine transaminase (*3hkt*) and hormone receptor 3 (*hr3*) RNAi vectors to remove antibiotic resistance marker genes and retain the expression cassette of the inverse repeat sequence of the *3hkt/hr3* target gene. This recombinant microalgal marker-free RNAi insecticide was subsequently added to the suburban water in a simulated-field trial to test its ability to control mosquito population.

**Methods:**

The expression cassette of the *3hkt/hr3* inverted repeat sequence and a DNA fragment of the argininosuccinate lyase gene without the ampicillin resistance gene were obtained using restriction enzyme digestion and recovery. After the cotransformation of *Chlamydomonas*, the recombinant algae was then employed to feed *Aedes albopictus* larvae. Ten and 300 larvae were used in small- and large-scale laboratory *Ae.albopictus* feeding trials, respectively. Simulated field trials were conducted using Meishe River water that was complemented with recombinant *Chlamydomonas*. Moreover, the impact of recombinant microalgae on phytoplankton and zooplankton in the released water was explored via high-throughput sequencing.

**Results:**

The marker-free RNAi-recombinant *Chlamydomonas* effectively silenced the *3hkt/hr3* target gene, resulting in the inhibition of *Ae. albopictus* development and also in the high rate of *Ae. albopictus* larvae mortality in the laboratory and simulated field trials. In addition, the results confirmed that the effect of recombinant *Chlamydomonas* on plankton in the released water was similar to that of the nontransgenic *Chlamydomonas*, which could reduce the abundance and species of plankton.

**Conclusions:**

The marker-free RNAi-recombinant *Chlamydomonas* are highly lethal to the *Ae. albopictus* mosquito, and their effect on plankton in released water is similar to that of the nontransgenic algal strains, which reduces the abundance and species of plankton. Thus, marker-free recombinant *Chlamydomonas* can be used for mosquito biorational control and mosquito-borne disease prevention.

**Graphical Abstract:**

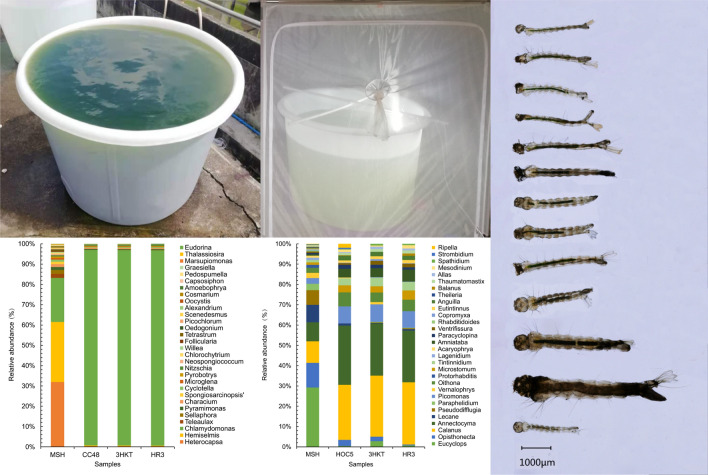

**Supplementary Information:**

The online version contains supplementary material available at 10.1186/s13071-022-05647-3.

## Background

Mosquitoes transmit several serious diseases, including dengue, dengue hemorrhagic fever, malaria, chikungunya, Zika, West Nile fever, Japanese encephalitis and yellow fever. Malaria alone was responsible for > 400,000 deaths worldwide in 2015, according to the World Health Organization estimates. Dengue fever, also a mosquito-borne disease, kills > 20,000 people each year worldwide. Climate change, globalization and viral evolution have all contributed to an increase in the prevalence of dengue and other mosquito-borne diseases [1, 2]. From 2000 until 2011, dengue fever in China was rare, but in the following year, it expanded swiftly. With 46,864 cases reported, there was a severe outbreak of dengue fever in China in 2014. From 2015 to 2018, the incidence of dengue fever followed a predictable and variable pattern, with a clear upward trend in 2019 with 22,599 reported cases [3]. The Flaviviridae family of viruses includes the Zika virus, which has been associated with Guillain-Barré syndrome, infant microcephaly as well as a feverish disease resembling dengue fever. The Togaviridae family virus, also known as the chikungunya virus, is diagnosed with fever that is characterized by severe chronic arthralgia [4, 5].

Utilizing pesticides to reduce mosquito populations is a crucial step in the prevention and management of diseases carried by mosquitoes. However, mosquitoes have a brief life cycle and a large number of offspring that allow them form succeeding generations with diverse genetic traits. This results in pesticide resistance [6, 7]. To lessen vector transmission, many vector control strategies, including the use of parasitic fungus and predatory fish, have been developed [8–11].

RNA interference (RNAi) technology refers to the silencing of target gene expression caused by the intervention of double-stranded RNA (dsRNA) [12–16]. This technology has been used in mosquito control recently [17–21]. RNase III-Dicer cleaves dsRNA into small interfering RNA (siRNA) of 20–25 nucleotides in the target cells. The siRNA is then put together by the Argonaute proteins to create the RNA-induced silencing complex (RISC), which subsequently destroys the endogenous mRNA complementary to its guide strand [22, 23]. dsRNA can currently be administered by immersion, oral feeding and microinjection [24–29]. The specificity of RNAi makes these methods environmentally safer than the chemical pesticides currently in use, thereby minimizing the toxicity to non-target species and reducing the likelihood of resistance to insect populations.

In *Ae. albopictus*, the hormone receptor 3 (*hr3*) gene plays an important role in metamorphosis. It is an important member of ecdysone signal transduction pathway [30]. In the tryptophan catabolism pathway, 3-hydroxykynurenine transaminase (3-HKT) catalyzes the conversion of 3-hydroxykynurenine (3-HK) to xanthurenic acid (XA) [31]. 3-HK is a highly reactive intermediate, which automatically oxidizes under normal physiological conditions to produce reactive oxygen that can kill insects [31]. These two genes have been identified as dsRNA silencing targets for mosquito population control [32, 33].

The biosafety of extensively genetically modified organisms has been a topic of public interest. As a result, it is critical to generate marker-free progenies in which the marker gene such as antibiotic resistance or herbicide inactivated genes used to generate positive transgenic organisms is removed [34, 35]. Marker-free transgenic organisms can be created using the following methods: flippase (FLP)/FLP recombinase target site-specific recombination [36, 37], Cre/lox site-specific recombination [38–41], multi-autotransformation [42], transcription activator-like effector nucleases [43–46], DNA-free gene editing based on the CRISPR/Cas system [47–49] and cotransformation [50, 51]; cotransformation is the most effective and straightforward of these methods [52]. The cotransformation technique has been used to create marker-free transgenic soya bean [53], tobacco [41], maize [45], rice [54], wheat [55, 56] and sorghum [57] in plants. For the genetic transformation of *Chlamydomonas reinhardtii*, cotransformation was utilized to study the promoter functions with arg7.8 in an arginine-deficient medium for the selection of transformants [58–61]. Cotransformation avoids potential threats to the environment that may be caused by the use of antibiotic-resistance marker genes.

In the present study, we modified the *3hkt* and *hr3* RNAi vectors, which are highly lethal to *Aedes* mosquitoes and were used in previous studies [32, 33], to remove antibiotic resistance marker genes on the vectors and retain the expression cassette of the inverse repeat sequence of the *3hkt/hr3* target gene. The modified RNAi vectors were then cotransformed with DNA fragments containing the argininosuccinate lyase (*asl*) gene into *C. reinhardtii* to obtain *3hkt/hr3* RNAi transgenic algae strains without antibiotic marker genes. In addition, the study explored the impact of recombinant microalgae on phytoplankton and zooplankton in the released waters using high-throughput sequencing to lay the foundation for the safe use and monitoring of recombinant microalgae.

## Methods

### Mosquito rearing

Mosquitoes were reared in accordance with our earlier research [32, 33]. In Haikou, China, wild *Ae. albopictus* mosquitoes were captured and brought to our laboratory. The mosquitoes were reared in an environment with a relative humidity of 70–80% and a temperature of 26 °C. To increase egg production, mature females were fed chicken blood, while adult males were raised in a 10% sugar solution. For growth, the larvae were fed rat meal.

### Algal strains and growth conditions

The University of Minnesota’s *Chlamydomonas* Resource Center provided *C. reinhardtii* CC48 (arg2 mt +), which was grown in TAP medium with 250 g of arginine per liter [57, 62]. To maintain liquid cultures at 25 °C, 150 µmol m^−2^ s^−1^ of steady light and 180 rpm shaking were used. Strains were incubated on TAP agar plates at 22 °C and 100 µmol m^−2^ s^−1^ [63]. *Chlamydomonas reinhardtii* CC48 was used as the recipient algae for RNAi expression framework transformation.

### Preparation of 3*hkt/hr3* RNAi expression cassette

**Fig. 1:**
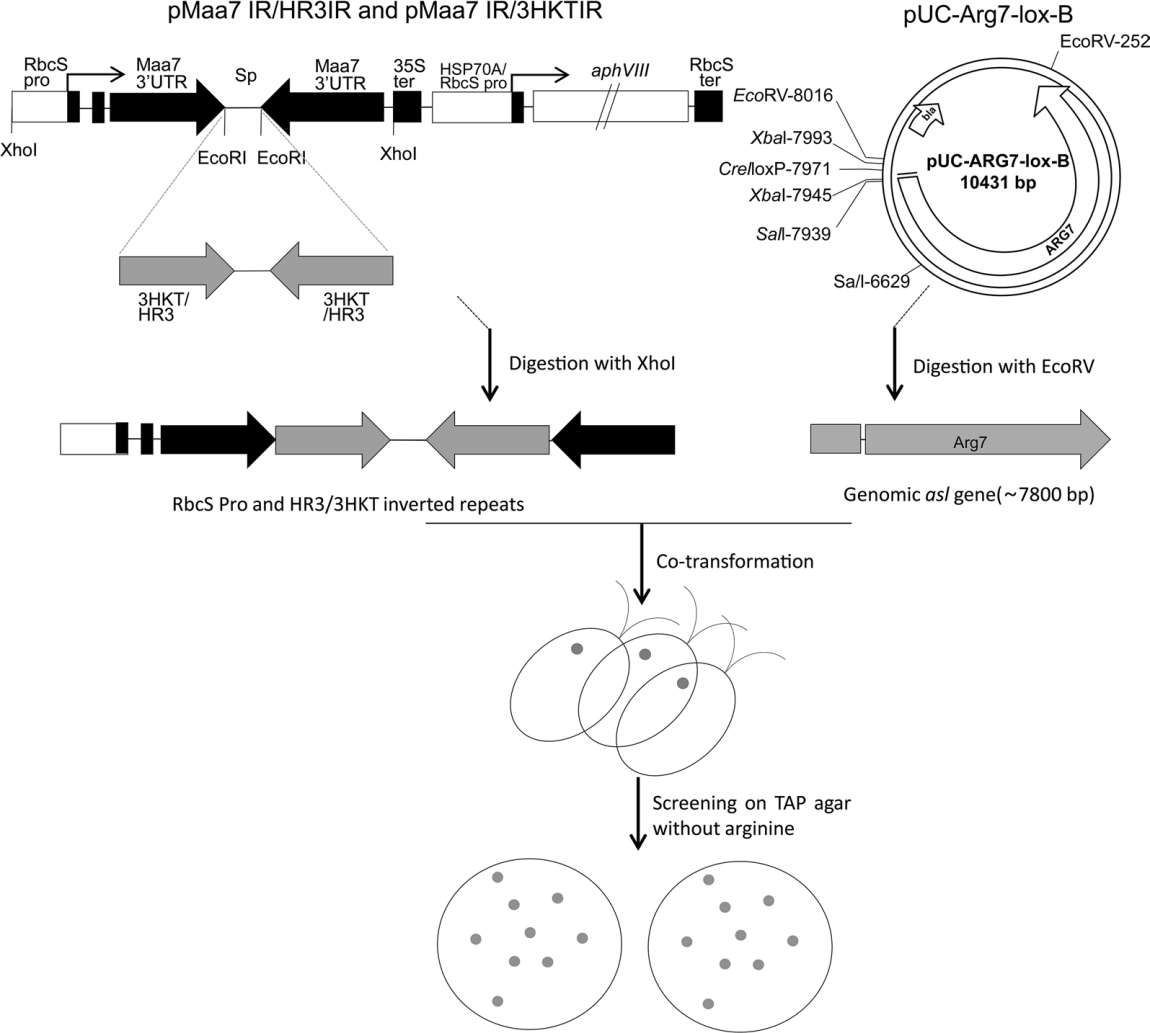
Schematic diagram of co-transformation strategy. pMaa7 IR/HR3IR and pMaa7 IR/3HKTIR were digested with XhoI, and the expression cassettes containing RbcS Promoter and HR3/3HKT inverted repeats were recovered by agarose gel electrophoresis. The plasmid pUC-Arg7-lox-B containing the genomic ARG7.8 gene was digested with EcoRV, and the fragment containing the argininosuccinate lyase (*asl*) gene was recovered. Through co-transformation, *Chlamydomonas* cells were plated on TAP agar without arginine until the algal colonies appeared 5–10 days later

The previously constructed RNAi recombinant plasmids pMaa7 IR/HR3IR and pMaa7 IR/3HKTIR were digested using *XhoI*, and the expression cassettes containing the RbcS promoter and *3hkt/hr3* inverted repeats were recovered via agarose gel electrophoresis [60, 61]. The plasmid pUC–Arg7–lox-B containing the genomic ARG7.8 was digested using *EcoRV*, and the fragment containing *asl* gene was recovered (Fig. 1) [64].

Through the above operations, the expression cassette of the *3hkt/hr3* inverted repeat with the aminoglycoside 3′-phosphotransferase (*aphviii*) gene was removed, and a DNA fragment of *asl* without the ampicillin resistance gene (*bla*) was obtained.

### Cotransformation

The expression cassette of the *3hkt/hr3* inverted repeat was introduced into the *Chlamydomonas* cells via co-transformation with the *asl* DNA fragment using the glass bead method [57, 65]. Cells were centrifuged and resuspended in arginine-free TAP medium. *Chlamydomonas* cells (400 ml), DNA (2–4 mg of the *3hkt/hr3* inverted repeat expression cassette, 4 mg of *asl* DNA fragment), 100 ml 20% polyethylene glycol and 300 mg sterile glass beads were mixed, and the mixture was vortexed for 15 s. After washing the cells to remove the glass beads, they were plated on the arginine-free TAP agar medium until algal colonies appeared.

### Mosquito feeding tests

The transgenic algae were subjected to polymerase chain reaction (PCR) analysis to verify the integration of the 3hkt/hr3 inverted repeat expression cassette into the genome of *Chlamydomonas* after the expression cassette of the 3hkt/hr3 inverted repeat that was introduced into *Chlamydomonas*. The positive transformants were then fed to the mosquitoes in the laboratory. As previously stated [61], mosquito feeding tests were conducted in the insectary with various groups of mosquitoes. Ten L1 larvae in each group were given 2.5 mg of fresh algae in 5 ml water. Larvae in the control groups were fed *Chlamydomonas* CC48, water and fodder, and those in the treated groups were fed recombinant *Chlamydomonas* strains (HR3-1 to HR3-4, 3HKT-1 to 3HKT-4) carrying 3hkt/hr3 RNAi expression cassettes. Larvae fed 1 mg dry powder of recombinant *Chlamydomonas* (HR3-D1 and 3HKT-D3) were the treatment group. The tests were carried out in triplicate. The lengths of three L3 larvae from each group was measured. Records were kept of the larval mortality, pupation and adult emergence rates. For further evaluation, the mosquitoes were treated with algae; 20 mg of fresh algae was fed to 300 L1 larvae kept in 50 ml water. Recombinant *Chlamydomonas*-fed larvae were referred to as the treatment group, while larvae given *C. reinhardtii* CC48, water and food were the controls. We measured the larval mortality and pupation rates as well as the adult emergence rates. Three duplicates of each experiment were carried out.

### Verification of mRNA in *Ae. albopictus*

Twenty to 30 L4 larvae were gathered and pooled together for qRT-PCR. The total RNA from the larvae was then isolated using the TRIzol Reagent (Takara). Using oligo-dT primers, single-stranded cDNA was synthesized from total RNA. SYBR green as the fluorescent dye was used, and real-time PCR was carried out on the BioRad iCycler iQ Real-Time PCR Detection System. Primers with the sequences 5′-aagaagtggccatcattcca-3′ and 5′-GGTCTCCGGGTCGACTTC-3′ were employed for the internal control of *Aedes RPS17* amplification [66]. Primer sequences 5′-gagcgatcaatatggccaccc-3′ and 5′-aatgggcgttattccaggtgg-3′ were used for *3hkt* quantification, while 5′-ATTTGCGCTAACATGCTATCG-3′ and 5′-CAGCCATTTCAAGTTCACTACG-3′ were used for *hr3* quantification. The PCR baseline subtracted method, carried out in the iCycler software at a constant fluorescence level, was used to determine the amplification rate of each transcript (Ct). The relative fold differences were computed using the relative quantification analytical method (2^−ΔΔCT^) [67].

### Analysis of water parameters and *Chlamydomonas* growth in the target water area

Water samples were collected from the Meishe River, Shapo Reservoir and Hongcheng Lake in Haikou City. The model HQ30d multiparameter meter (HACH, China) was used to measure nitrogen, phosphorus, ammonia nitrogen, nitrite nitrogen, nitrate nitrogen, chemical oxygen demand (COD) and silicate levels in the samples. The water samples were centrifuged at 5000 rpm for 5 min, and the algal species thus collected were observed and identified under the microscope. Subsequently, 30 ml 5 × 10^6^ *Chlamydomonas* was inoculated into 10 l of the water samples, and the growth kinetics of *Chlamydomonas* were observed and recorded.

### Simulated field trials

Four mosquito breeding cages of 5 m^3^ volume made up of 0.4-mm aperture polyester mesh were prepared according to the method of Mysore et al. with modifications [68]. Four 1000-l buckets were placed in the cages for microalgae culture. Light-emitting diode bulbs were installed in the cage to enable algal photosynthesis in the water, and a ventilation pump was used for continuous water circulation. Algae were cultured in the buckets until their concentration gradually expanded to the logarithmic phase in the 100 l medium; 700 l of Meishe River water was added to this medium to adjust the volume of algal liquid in the barrel to 800 l. The ventilation pump was subsequently turned on, and the pressure of the pump was adjusted to enable water flow in the barrel. Approximately 1000 L1 larvae were then placed in each cage. Male adults were provided with a 10% sucrose solution, whereas female adults were given chicken blood for egg-laying. The number of adult *Aedes* mosquitoes was counted once every week.

### Sample preparation and DNA extraction of test water

To detect the effect of recombinant *Chlamydomonas* on the plankton in the test water, 18S high-throughput DNA sequencing analysis was performed on the water sample in the simulated field trials. A 1-l sample of the bucket's water was taken after the *Aedes* mosquitoes had been fed for 28 days. Plankton was extracted using a 0.40-μm polycarbonate membrane (Millipore, USA) at a vacuum pressure of 30 kPa. The membranes were kept until analysis at 80 °C. The samples' genomic DNA was then extracted [69].

### High-throughput sequencing

The target area for sequencing was the 18S rDNA’s V4 hypervariable region. A pair of universal primers D514 and B706R were used to amplify the target area [70, 71]. Then, following the manufacturer’s instructions, with the use of the NEB library preparation kit, sequence libraries were produced (Illumina, USA). After the library’s quality had been assessed, the Illumina HiSeq2500 platform was then used to sequence the libraries. Operational taxonomic units (OTUs) were formed from the sequences that shared > 97% similarities. The NCBI nucleotide and Silva databases were used in the taxonomic assignment of OTUs to obtain accurate results [72–74].

### Statistical evaluations

SPSS25 was used to analyze the data. The data are presented as the mean and standard deviation. To examine significant differences between means, Duncan’s multiple range test and Student’s t-test were used. Error bars show standard deviation, while asterisks denote statistical significance: *P < 0.05 and **P < 0.01, respectively.

## Results

### Recombinant *Chlamydomonas* with a *3hkt/hr3 *RNAi expression cassette are fatal to *Ae. albopictus*.

As RNAi target genes, *3hkt* and *hr3* (GenBank: XM 021849682, AF230281) were employed. In the coding sequence, the *3hkt* target region for RNAi silencing was located between 329 and 648, whereas the *hr3* target region was located between 263 and 537. The expression cassettes containing *3hkt/hr3* inverted repeats were recovered and cotransformed with the genomic *ARG7.8* gene digested using *EcoRV* after the RNAi expression plasmids pMaa7 IR/HR3IR and pMaa7 IR/3HKTIR had been digested using *XhoI* (Fig. 1). PCR was utilized to positively identify > 100 altered algal strains, which were then employed in subsequent tests.

**Fig. 2:**
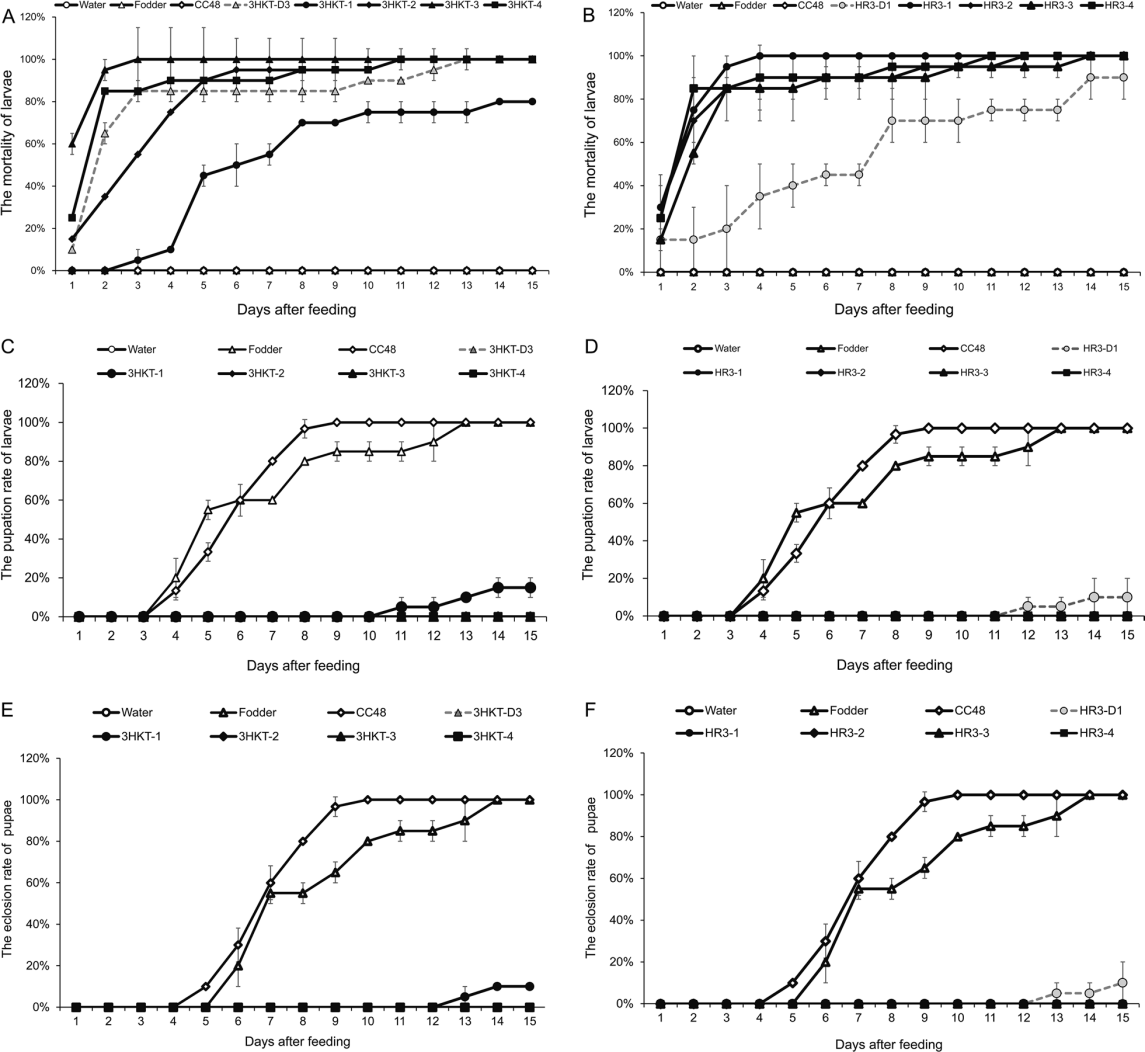
*Aedes albopictus* mortality (**A**, **B**), pupation (**C**, **D**) and eclosion rate (**E**, **F**) when fed recombinant *Chlamydomonas*. Water: water is fed to the larvae; CC48: larvae fed *C. reinhardtii* CC48; fodder: larvae fed fodder; 3HKT-1 to 3HKT-4: larvae fed with 3HKT RNAi expression cassette co-transformation *Chlamydomonas* strains 3HKT-1 to 3HKT-4; HR3-1 to HR3-4: larvae fed with HR3 RNAi expression cassette co-transformation *Chlamydomonas* strains HR3-1 to HR3-4. HR3-D1 and 3HKT-D3: larvae fed with inactive dry powder of recombinant *Chlamydomonas* HR3-1 and 3HKT-3, respectively. The experiment was done three times, and the average values are presented. Each treated and control group contained ten *Aedes* larvae. Time frame: 15 days

The larvae fed with recombinant *Chlamydomonas* strains (3HKT2 to 3HKT4, HR3-1 to HR3-4) began dying on the second day in laboratory experiments. All larvae fed with recombinant strains died in 15 days, with the exception of 3HKT1 and HR3-D1. However, when given water, food and *C. reinhardtii* CC48, none of the larvae perished within 15 days. These findings showed that oral administration of transgenic *Chlamydomonas* bearing the *3hkt/hr3* RNAi expression cassette is fatal to *Aedes* larvae (Fig. 2A, B). Regarding the pupae formation of *Aedes* mosquitoes, only 15% and 10% of larvae fed with the recombinant *Chlamydomonas* 3HKT1 and HR3-D1, respectively, pupated, and none of the larvae were fed with the other recombinant *Chlamydomonas* strains pupated.

**Fig. 3:**
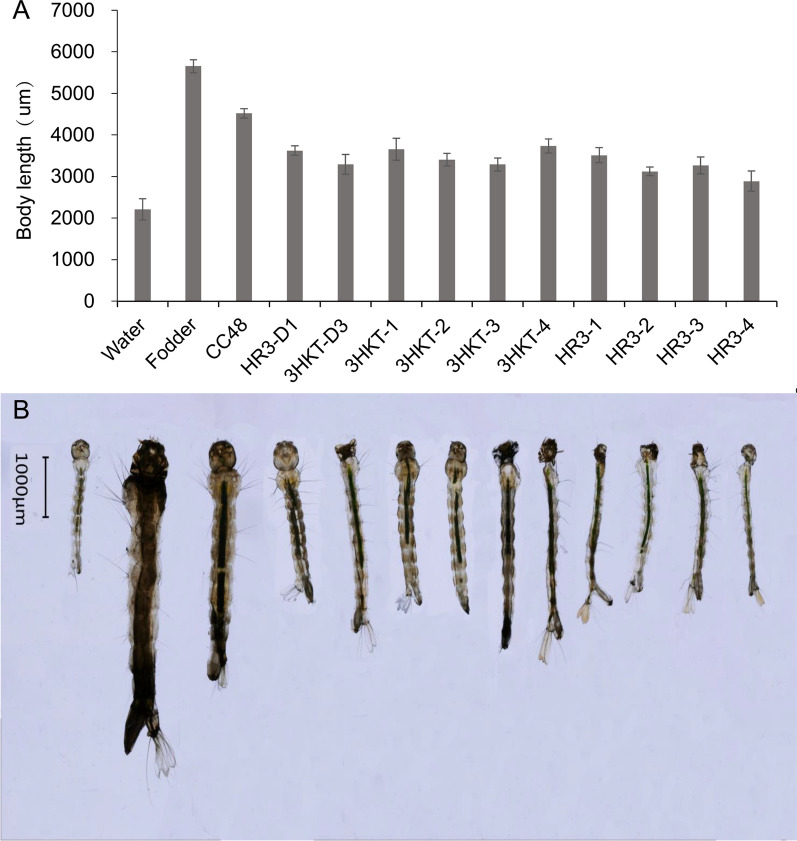
Length of larvae. The length of L3 larvae from each treatment was measured. Water: water is fed to the larvae; CC48: larvae fed *C. reinhardtii* CC48; fodder: larvae fed fodder; 3HKT-1 to 3HKT-4: larvae fed with 3HKT RNAi expression cassette co-transformation *Chlamydomonas* strains 3HKT-1 to 3HKT-4; HR3-1 to HR3-4: larvae fed with HR3 RNAi expression cassette co-transformation *Chlamydomonas* strains HR3-1 to HR3-4. HR3-D1 and 3HKT-D3: larvae fed with inactive dry powder of recombinant *Chlamydomonas* HR3-1 and 3HKT-3, respectively. Data are expressed as mean ± SD (*n* = 3), and significant differences (*P* < 0.05, Duncan’s multiple range tests) are shown by different letters

In the control groups, 100% of larvae fed with fodder or *C. reinhardtii* CC48 pupated, whereas none of the larvae pupated which were fed with water (Fig. 2C, D). In terms of the adult eclosion of *Aedes* mosquito, other than the 15% and 10% larvae fed with recombinant *Chlamydomonas* 3HKT1 and HR3-D1 that eclosed into adults, all pupae fed with recombinant *Chlamydomonas* did not eclose into adults. However, 100% of pupae from the control groups that were fed with fodder and *C. reinhardtii* CC48 developed into adults (Fig. 2E, F). The *Aedes* mosquitoes fed with fodder had the longest L3 larval body length (5.7 mm), followed by mosquitoes fed with *C. reinhardtii* CC48, with a body length of 4.5 mm. The body length of mosquitoes fed with water was the shortest (2.2 mm), whereas the body length of other mosquitoes fed with recombinant strains was significantly lower than that of the control group (fed with fodder and *C. reinhardtii* CC48) (Fig. 3A, B).

3HKT-D3 and HR3-D1 were the inactivated dry powders of recombinant *Chlamydomonas* 3HKT-3 and HR3-1, respectively. The lethal effect of 3HKT-D3 and HR3-D1 on *Aedes* mosquitoes was lower than that of 3HKT-3 and HR3-1; however, the lethal effect was maintained. This result is useful in the context of the commercial application of this biopesticide technology.

### *Aedes albopictus* feeding experiment

**Fig. 4:**
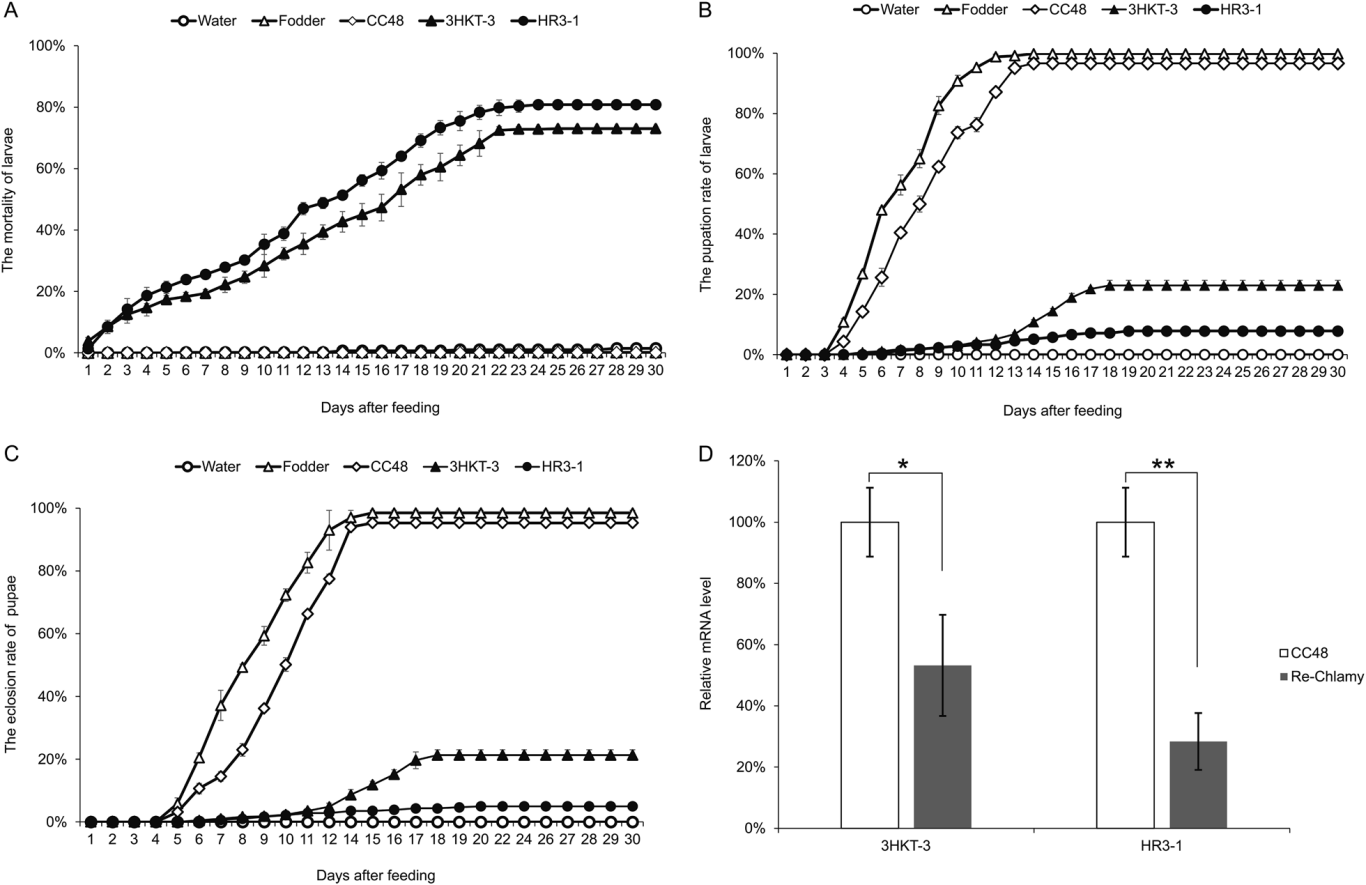
Mortality (**A**), pupation (**B**) and eclosion rate (**C**) of *Ae. albopictus* fed with the recombinant *Chlamydomonas* and the relative 3HK/HR3 mRNA levels in *Aedes* L4 larvae fed with recombinant *Chlamydomonas* (**D**). Water: water is fed to the larvae; CC48: larvae fed *C. reinhardtii* CC48; fodder: larvae fed fodder; 3HKT-3: larvae fed with 3HKT RNAi expression cassette co-transformation *Chlamydomonas* strains 3HKT-3; HR3-1: larvae fed with HR3 RNAi expression cassette co-transformation *Chlamydomonas* strains HR3-1. Re-Chlamy: larvae fed with recombinant *Chlamydomonas*

In this experiment, approximately 300 L1 *Ae. albopictus* larvae in each treatment group were tested for 30 days. The larvae fed with recombinant *Chlamydomonas* 3HKT-3 and HR3-1 started dying on the 2nd day, and 73.00% and 80.83% of the larvae, respectively, died within 30 days. By contrast, only 1.50%, 0.17% and 0.00% of larvae fed with water, fodder and *C. reinhardtii* CC48, respectively, died (Fig. 4A).

On the 4th day, the larvae fed with fodder started to pupate, and 99.83% of them did so within 30 days. On the 4th day, the larvae fed with *C. reinhardtii* CC48 also started to pupate; 96.67% of them did so within 30 days. The larvae fed with the recombinant *Chlamydomonas* 3HKT-3 and HR3-1 began pupating on the 5th and 6th day, respectively, and only 23.00% and 7.83% larvae, respectively, pupated by 30 days (Fig. 4B).

Pupae fed with fodder fully developed into adults on the 15th day, with 98.50% developing into adults within 30 days. Pupae fed *C. reinhardtii* CC48 reached adulthood on the 15th day, with 95.33% reaching adulthood within 30 days. Only 21.33% and 5.00% of pupae fed with recombinant *Chlamydomonas* 3HKT-3 and HR3-1 emerged as adults, respectively (Fig. 4C). qRT-PCR was used to examine the expression of *3hkt/hr3* in *Aedes* larvae and found that the expression level of *3hkt* in larvae fed with recombinant *Chlamydomonas* 3HKT-3 was 47% lower than in the control (larvae fed with C. reinhardtii CC48). The level of *hr3* expression in larvae fed with recombinant *Chlamydomonas* HR3-1 was 72% lower compared to control larvae (Fig. 4D). These results suggest that recombinant *Chlamydomonas* effectively silences *3hkt* and *hr3* in *Aedes* mosquitoes.

### Simulated field evaluation of the activity of recombinant *Chlamydomonas* 3HKT-3 and HR3-1

In preparation for future field studies, the activity of recombinant *Chlamydomonas* 3HKT-3 and HR3-1 was assessed under simulated field conditions. First, the quality of water collected from three areas in Haikou City was tested to detect whether *Chlamydomonas* CC48 can suitably grow in the water bodies. The results revealed that the nitrogen, phosphorus, ammonia, nitrate, nitrite and COD levels in the water samples of the Meishe River, Shapo Reservoir and Hongcheng Lake exceeded their respective upper limits in clean water, indicating pollution and eutrophication in the water bodies. In addition, the water salinity of Hongcheng Lake reached 1.5% (Additional file 5: Table S1). The results indicated that owing to eutrophication, the water of the Meishe River and Shapo Reservoir could support the growth of *Chlamydomonas*. However, because of the high salinity, the water of Hongcheng Lake was not suitable for the growth of *Chlamydomonas*.

We used 10 l water from the above three areas to culture recombinant *Chlamydomonas* 3HKT-3 and HR3-1. *Chlamydomonas reinhardtii* CC48 could grow normally in the waters of the Meishe River and Shapo Reservoir and reached the peak on the 7th day. However, due to high salinity (1.5%) of the water from Hongcheng Lake, *C. reinhardtii* CC48 could not grow normally (Additional file 1: Fig. S1). Considering that the location of the simulated field trial was near the water intake of the Meishe River, its water was used for the trial.

**Fig. 5:**
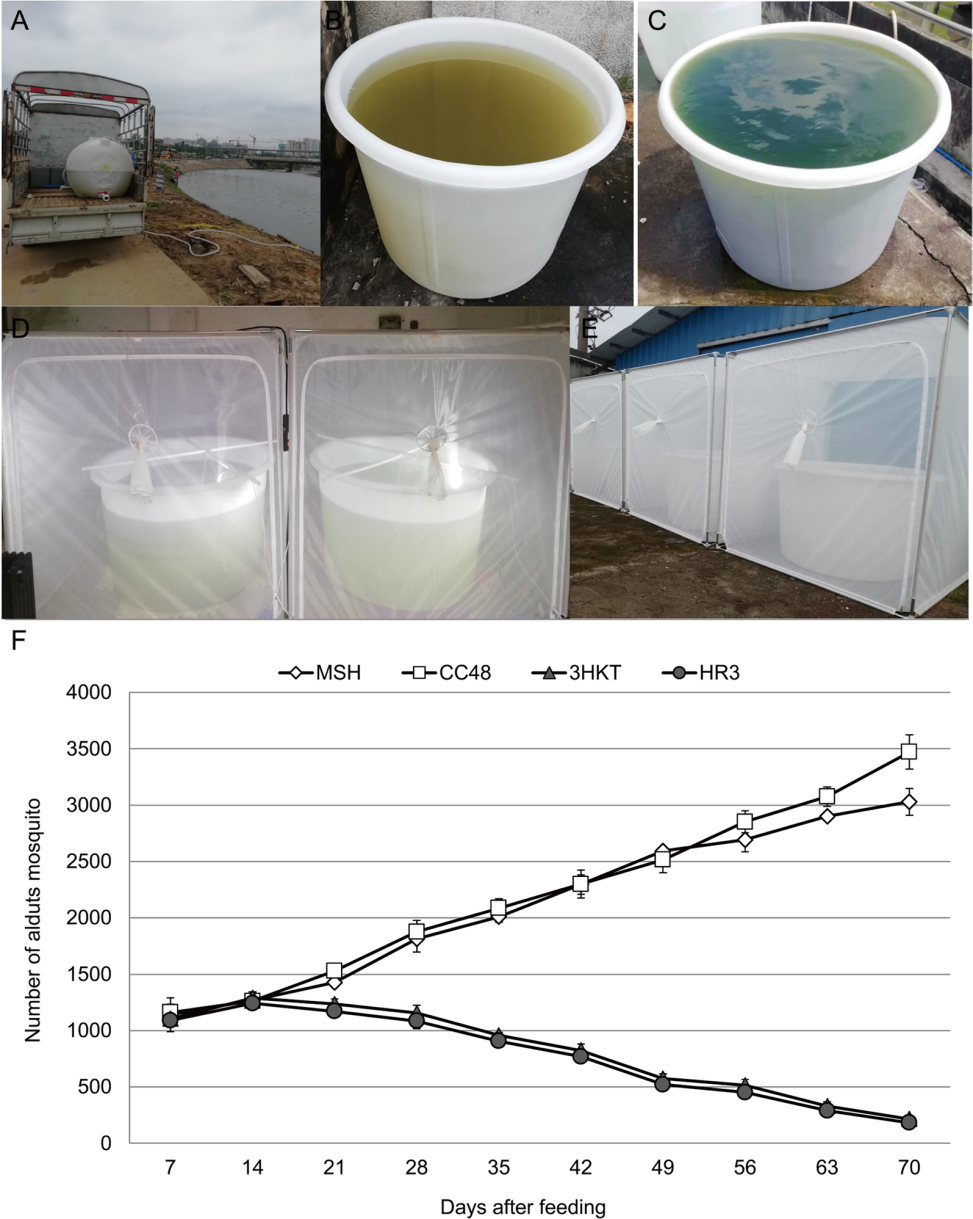
Simulated field tests in a Haikou neighborhood. **A** and **B** Meishe River was used for the test water. **C** 700 l Meishe River water and 100 l *Chlamydomonas* were combined in the barrel. **D** and **E** Each cage contained about 1000 pupae, and every week the number of adult mosquitoes was counted. **F** *Aedes albopictus* population and survival rates in MSH, CC48, 3HKT and HR3 treatments. MSH: During this treatment, mosquitoes exclusively drank water from the Meishe River. 3HKT and HR3: In this treatment, mosquitoes were kept in water from the Meishe River that had been supplemented with recombinant *Chlamydomonas* 3HKT-3 and HR3-1, respectively. CC48: In this treatment, mosquitoes were kept in water from the Meishe River that had been supplemented with *C. reinhardtii* CC48

To reduce the impact on residents, an idle factory building in the suburbs of Haikou City, 5 km away from the nearest residential area, was selected for the trials (Fig. 5A–E). The results revealed that the number of *Aedes* mosquitoes fed with the water of the Meishe River increased from 1140 to a maximum of 3029 individuals after 70 days, and the number of *Aedes* mosquitoes fed with the water of the Meishe River supplemented with *C. reinhardtii* CC48 increased to a maximum of 3470 individuals after 70 days. In the treatment groups, *Aedes* mosquitoes fed with the water of the Meishe River supplemented with recombinant *Chlamydomonas* 3HKT-3 and HR3-1 decreased from 1104 and 1089 to 215 and 182, respectively (Fig. 5F). These results suggested that recombinant *Chlamydomonas* 3HKT-3 and HR3-1 reduced the *Aedes* mosquito population under simulated field conditions.

### Sequencing results of 18S V4 hypervariable region in the test water

To understand the impact of recombinant *Chlamydomonas* on the biological population in the test water, an 18S high-throughput DNA sequencing analysis was performed. After quality control, a total of 80,986 qualified tags were identified.

After removing unclassified and unique tags from the dataset, the total number of high-quality tags were 72,486 with 182 assigned OTUs (Additional file 2: Fig. S2).

**Fig. 6:**
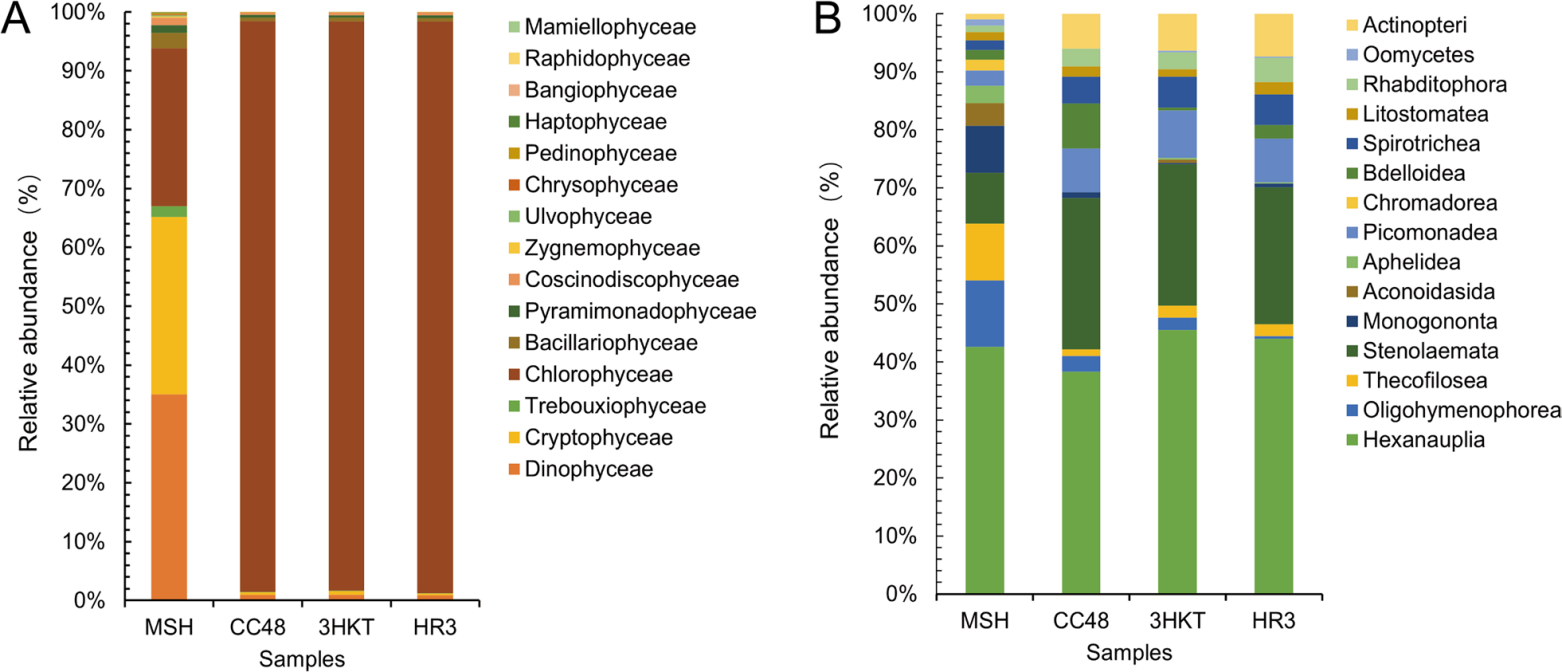
Variation in the class level of phytoplankton (**A**) and zooplankton (**B**) relative abundance in test waters from MSH, CC48, 3HKT and HR3. MSH: During this treatment, mosquitoes exclusively drank water from the Meishe River. 3HKT and HR3: In this treatment, mosquitoes were kept in water from the Meishe River that had been supplemented with recombinant *Chlamydomonas* 3HKT-3 and HR3-1, respectively. CC48: In this treatment, mosquitoes were kept in water from the Meishe River that had been supplemented with *C. reinhardtii* CC48

The Meishe River group (MSH) had a total of 15 groups of eukaryotic microalgae(class level), including *Chlorophyceae*, *Trebouxiophyceae*, *Dinophycea*, *Cryptophyceae*, *Bacillariophyceae*, *Coscinodiscophyceae*, *Pyramimonadophyceae*, *Zygnemophyceae*, *Ulvophyceae*, *Chrysophyceae*, *Pedinophyceae*, *Haptophyceae*, *Bangiophyceae*, *Raphidophyceae* and *Mamiellophyceae* (Fig. 6A). Among these groups, 65 OTUs within 45 genera were identified in the MSH group. *Chlorophycae* contained the most OTUs, 20 of which were spread over its 17 genera. The second-largest class in terms of OTU diversity was *Dinophycea*. Within four genera, eight OTUs were present. Additionally, a sizable number of OTUs were found in *Bacillariophyceae*, *Trebouxiophyceae* and *Cryptophyceae*. Comparatively fewer OTUs were present in the *Bangiophyceae*, *Raphidophyceae* and *Mamiellophyceae* classes (Additional file 3: Fig. S3).

The CC48 group (Meishe River plus *C. reinhardtii* CC48) had a total of 14 groups of eukaryotic microalgae (class level), including *Chlorophyceae*, *Dinophyceae*, *Cryptophyceae*, *Trebouxiophyceae*, *Bacillariophyceae*, *Pyramimonadophyceae*, *Coscinodiscophyceae*, *Zygnemophyceae*, *Chrysophyceae*, *Pedinophyceae*, *Haptophyceae*, *Bangiophyceae*, *Raphidophyceae* and *Mamiellophyceae* (Fig. 6A)*.* Among these, 55 OTUs within 41 genera were identified in the CC48 group. The most OTUs were found in *Chlorophycae*, which contained 16 OTUs in 15 genera (Additional file 3: Fig. S3). The second-largest class was *Dinophyceae*, which had eight OTUs in four genera. OTUs were mostly hosted by the *Cryptophyceae*, *Trebouxiophyceae* and *Bacillariophyceae* classes. Comparatively fewer OTUs were present in the *Bangiophyceae*, *Raphidophyceae* and *Mamiellophyceae* classes (Additional file 3: Fig. S3).

In the 3HKT-3 treatment group (Meishe River plus recombinant *Chlamydomonas* 3HKT-3), a total of 13 types of eukaryotic microalgae (class level) have been included, i.e. *Chlorophyceae*, *Dinophyceae*, *Cryptophyceae*, *Trebouxiophyceae*, *Bacillariophyceae*, *Pyramimonadophyceae*, *Coscinodiscophyceae*, *Zygnemophyceae*, *Ulvophyceae*, *Chrysophyceae*, *Pedinophyceae*, *Haptophyceae* and *Raphidophyceae* (Fig. 6A). Among these, 54 OTUs within 40 genera were identified in the 3HKT group. The most OTUs were found in *Chlorophycae*, which contained 16 OTUs in 15 genera. OTUs were mostly hosted by the following classes—*Dinophyceae, Cryptophyceae, Trebouxiophyceae* and *Bacillariophyceae*. Comparatively fewer OTUs were present in the *Zygnemophyceae, Ulvophyceae, Chrysophyceae, Pedinophyceae, Haptophyceae* and *Raphidophyceae* classes (Additional file 3: Fig. S3).

A total of 12 groups of eukaryotic microalgae were identified at the class level in the Meishe River plus recombinant *Chlamydomonas* HR3-1 treatment group (Fig. 6A). Among these, 56 OTUs within 40 genera were identified in the HR3 group. *Chlorophyceae* had the largest number of OTUs, with 17 OTUs within 15 genera. *Dinophyceae*, *Trebouxiophyceae*, *Cryptophyceae* and *Bacillariophyceae* hosted a relatively large number of OTUs, whereas *Zygnemophyceae*, *Ulvophyceae*, *Chrysophyceae*, *Haptophyceae* and *Raphidophyceae* had a relatively lower number of OTUs (Additional file 3: Fig. S3).

### Variation in the phytoplankton community in the test water

**Fig. 7:**
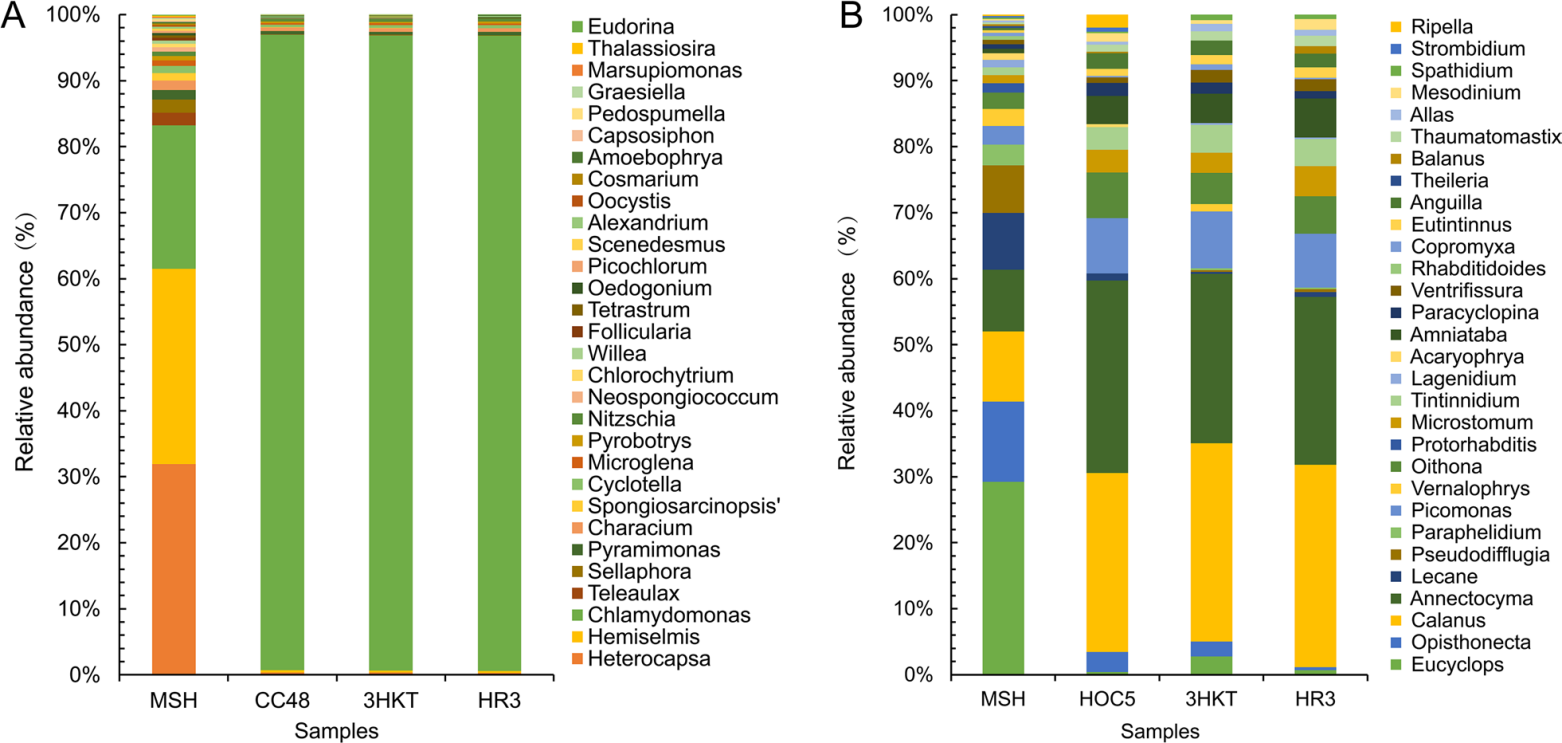
Variations in the relative abundance of phytoplankton (**A**) and zooplankton (**B**) species at the genus level in test waters from MSH, CC48, 3HKT and HR3. MSH: During this treatment, mosquitoes exclusively drank water from the Meishe River. 3HKT and HR3: In this treatment, mosquitoes were kept in water from the Meishe River that had been supplemented with recombinant *Chlamydomonas* 3HKT-3 and HR3-1, respectively. CC48: In this treatment, mosquitoes were kept in water from the Meishe River that had been supplemented with *C. reinhardtii* CC48

**Fig. 8:**
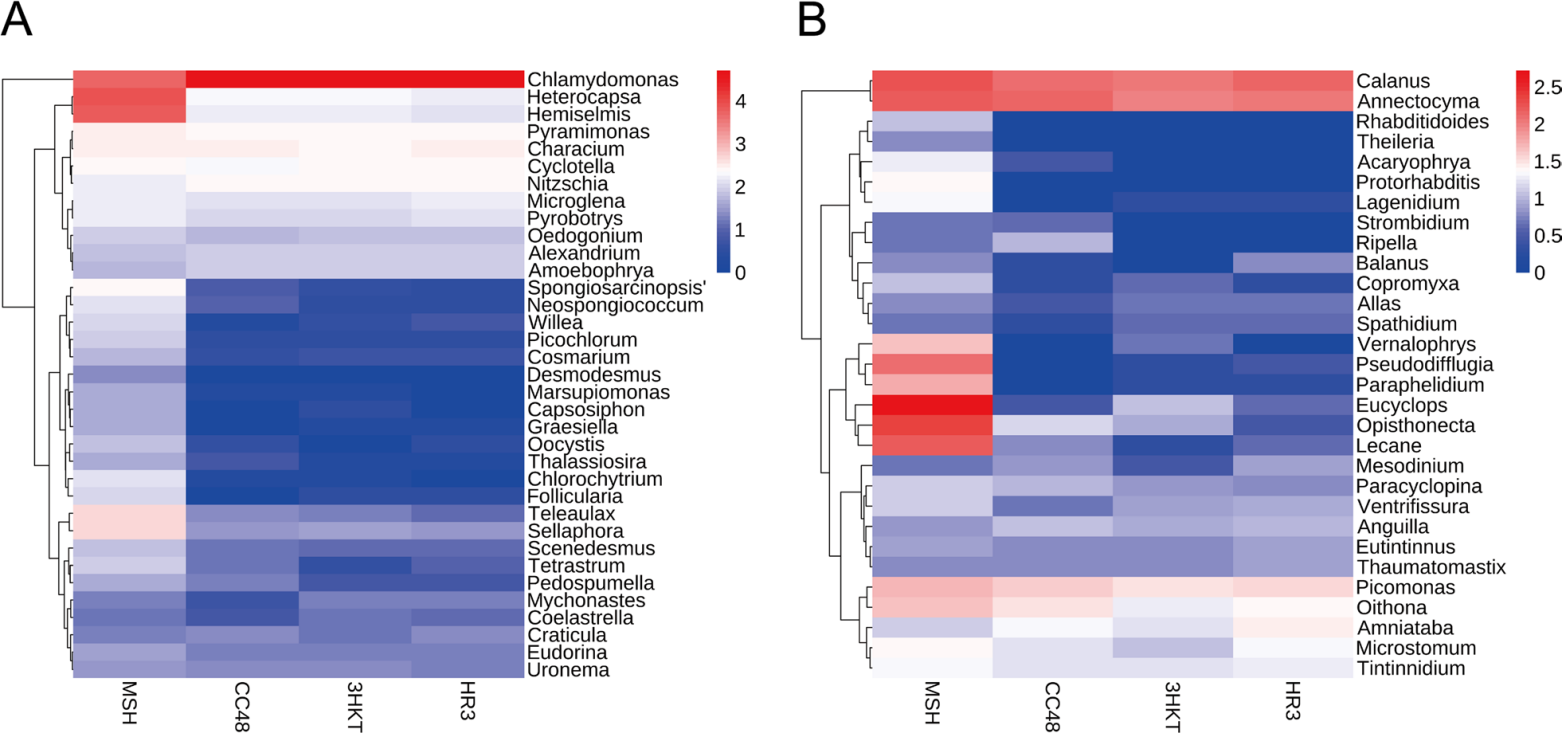
In test waters from MSH, CC48, 3HKT and HR3, phytoplankton (**A**) and zooplankton (**B**) were analyzed using a heat map. Sample names are indicated in the heat map’s horizontal ordinate at the bottom, and distinct phytoplankton or zooplankton classes are indicated in the ordinate on the right side. When values fall below the mean, the heat map’s hue is negative; when they rise, it is positive. The standard score (*Z*-values), which is represented by the color scale in the top right corner, is equal to (*x* − µ)/σ, where *x* represents the relative abundance of a particular plankton group. The average relative abundance of all plankton groups is known as μ. The standard deviation of relative abundance for all plankton groups is σ. For hierarchical clustering, the Bray-Curtis distance was determined using R software. MSH: During this treatment, mosquitoes exclusively drank water from the Meishe River. 3HKT and HR3: In this treatment, mosquitoes were kept in water from the Meishe River that had been supplemented with recombinant *Chlamydomonas* 3HKT-3 and HR3-1, respectively. CC48: In this treatment, mosquitoes were kept in water from the Meishe River that had been supplemented with *C. reinhardtii* CC48

The MSH group had high species diversity. When excess *C. reinhardtii* CC48 was added to the Meishe River water, *Chlamydomonas* abundance increased from 29.34% to 96.05% (CC48 group). In the treatment groups 3HKT and HR3, *Chlamydomonas* abundance rose to 95.98% and 95.99%, respectively (Additional file 4: Fig. S4). Because of the rapid increase in *Chlamydomonas* abundance, compared to the MSH group, the number of phytoplankton in the CC48 group decreased by four at the genus level and the number of OTUs decreased by seven, whereas the number of phytoplankton in the 3HKT and HR3 group decreased by five for both at the genus level and the number of OTUs decreased by eight and six, respectively (Fig. 7A, Additional file 6: Table S2). Compared with the MSH group, except for *Chlamydomonas*, *Nitzschia*, *Alexandrium* and *Amoebophrya*, the abundance of other major species decreased in the CC48, 3HKT and HR3 groups (Figs. 7A, 8A). These results suggest that when nontransgenic *Chlamydomonas* is added to the CC48 group or when recombinant *Chlamydomonas* is added to the 3HKT or HR3 group, the rapid increase in *Chlamydomonas* abundance leads to increased competition and consumption of nutrients in the water body, resulting in the death or reduction of the abundance of other algae because of nutrient deficiency.

### Variation in the zooplankton community in the test water

A total of 15, 12, 13 and 13 groups of protozoa, respectively, were identified at the class level in the MSH group, CC48 group, 3HKT group and HR3 group, respectively. *Hexanauplia*, *Oligohymenophorea*, *Thecofilosea*, *Stenolaemata*, *Monogononta*, *Aconoidasida*, *Aphelidea*, *Picomonadea*, *Chromadorea*, *Bdelloidea*, *Spirotrichea*, *Litostomatea*, *Rhabditophora*, *Oomycetes* and *Actinopteri* (Fig. 6B) were the main species. Among these, 29 OTUs within 26 genera were revealed in the MSH group, 24 OTUs within 20 genera in the CC48 group, 23 OTUs within 20 genera in the 3HKT group and 23 OTUs within 21 genera in the HR3 group (Additional file 6: Table S2). In contrast to the zooplankton species in the MSH group, the number of zooplankton species in the CC48 group decreased by six at the genus level and the number of OTUs decreased by five, whereas the number of zooplankton species in the 3HKT and HR3 groups decreased by six and five at the genus level and the number of OTUs decreased by eight for both, respectively (Fig. 7B, Additional file 6: Table S2). Compared with the MSH group, the abundance of major species decreased in the CC48, 3HKT and HR3 groups, except for *Calanus*, *Annectocyma*, *Amniataba* and *Anguilla* (Figs. 7B, 8B). These results suggest that the additions of both the nontransgenic *Chlamydomonas* in the CC48 group and recombinant *Chlamydomonas* in the 3HKT and HR3 groups decrease zooplankton abundance and species in the released water.

## Discussion

*Aedes* mosquitoes are the vectors of epidemic diseases that affect public safety worldwide. These diseases include yellow fever, dengue fever, Zika virus disease and chikungunya [75–78]. These diseases lead to a large number of deaths every year. The main method used to stop the spread of these diseases is vector control because there are no viable treatments or vaccinations. Numerous studies have demonstrated the effectiveness of RNAi in reducing insect populations, and it may be less vulnerable to insect resistance than to other methods. The public response to transgenic technology has been negative. The primary concern of the public is that antibiotic resistance marker genes may be potentially harmful to the human body. In addition, there is a concern about ecological safety after transgenic organisms are released into the environment.

The goal of the present study was to develop a low-cost and fast-acting biopesticide, which should ideally have the ability to reproduce itself to reduce production costs that can be released directly into suburban water bodies. Therefore, we modified *3hkt* and *hr3* RNAi vectors by eliminating the antibiotic resistance marker genes on the vectors and retaining the inverse repeat sequence of the *3hkt/hr3* target gene, which was obtained by cotransforming *C. reinhardtii* CC48 with the DNA fragment of *asl*.

The study results revealed that *Ae. albopictus* larvae fed with recombinant *Chlamydomonas* died from the 2nd day onward and all larvae fed with recombinant *Chlamydomonas* died within 15 days, except 3HKT1 and HR3-D1 (Fig. 2A, B). In the feeding experiment of 300 *Aedes* mosquitoes, 73.00% and 80.83% of the larvae fed with recombinant *Chlamydomonas* 3HKT-3 and HR3-1, respectively died within 30 days (Fig. 4A). These results indicate that *3hkt* and *hr3* RNAi expression cassette-containing recombinant *Chlamydomonas* administered orally is lethal to *Ae. albopictus* larvae.

Of note, we found that the lethal biological activity of dead recombinant *Chlamydomonas* against *Aedes* mosquitoes persisted. 3HKT-D3 and HR3-D1, the inactivated dry powders of recombinant *Chlamydomonas* 3HKT-3 and HR3-1, had a lower lethal effect on *Aedes* mosquitoes compared to 3HKT-3 and HR3-1; however, the lethal effect persisted (Fig. 2A, B). This result is similar to that found in yeast by Mysore et al. [68].

Considering that the main vector of the dengue virus in Hainan, China, was *Ae. albopictus*, this vector was chosen in this study [79, 80]. We selected the waters of the Meishe River in Haikou City to release recombinant *Chlamydomonas* because *Chlamydomonas* was one of the dominant algae strains in the water body (Figs. 7A, 8A, Additional file 4: Fig. S4A). In addition, the eutrophication of the Meishe River, with excessive nitrogen, phosphorus, ammonia and COD levels (Additional file 5: Table S1), was favorable for the survival and reproduction of recombinant *Chlamydomonas* (Additional file 1: Fig. S1).

The recombinant *Chlamydomonas* 3HKT-3 and HR3-1 reduced the number of *Aedes* mosquitoes from 1104 and 1089 to 215 and 182, respectively, within 70 days, and the *Ae. albopictus* population was effectively suppressed (Fig. 5F). To understand the impact of recombinant *Chlamydomonas* on the biological population of the test water, an 18S high-throughput DNA sequencing analysis was performed, which confirmed the rapid reproduction of *Chlamydomonas*. This phenomenon is similar to that of algal blooms in the river or lake, wherein nutrients in the water are mainly consumed by *Chlamydomonas*, leading to the decline of species abundance and type of other phytoplankton (Figs. 7A, 8A). The proliferation of *Chlamydomonas* also led to a decrease in the number of zooplankton species and abundance, thus suggesting that the massive reproduction of recombinant *Chlamydomonas* has an inhibitory effect on the protozoa population (Figs. 7B, 8B).

The study also confirmed that the effect of recombinant *Chlamydomonas* on plankton in water is similar to that of the nontransgenic *Chlamydomonas*, which can reduce plankton abundance and species in the water body.

Although some progress has been made in this study, a recombinant *Chlamydomonas* carrying an RNAi expression cassette was used to suppress the *Aedes* mosquito population, thereby reducing their chances of transmitting infectious agents. However, the impact of recombinant *Chlamydomonas* on environmental organisms, including those in the direct and indirect food chains, needs further evaluation. The aim of the present study was to develop a low-cost, environmentally friendly mosquito control technology, which was achieved using self-propagating microalgal insecticides. However, the effects of blooms caused by recombinant *Chlamydomonas* after its release into water bodies should be monitored. Recombinant *Chlamydomonas* should be maintained at levels that can control mosquito populations and have little impact on other plankton, making it acceptable to the public. Our research also needs to be continuously improved with the use of in vitro gene editing technology, shRNA technology and others to minimize the impact of exogenous DNA fragments harbored by recombinant microalgae.

## Conclusions

The present study indicated that the marker-free RNAi-recombinant *Chlamydomonas* are highly lethal to the *Ae.albopictus* mosquito, and its effect on plankton in released water is similar to that of the nontransgenic algal strains, which reduces the abundance and species of plankton. However, recombinant *Chlamydomonas* should be kept at a level that can control mosquito population and have little impact on other plankton so as to make it acceptable to the public. In this way, the marker-free recombinant *Chlamydomonas* is expected to be used for mosquito biorational control and mosquito-borne disease prevention.

## Supplementary Information

**Additional file 1: Figure S1.** The growth curve of *Chlamydomonas* CC48 in the water from Shapo Reservoir, Hongcheng Lake and Meishe River.

**Additional file 2: Figure S2.** Rarefaction curves for all samples taken from the test waters' operational taxonomic units (OTUs). Weak slopes at the end of rarefaction curves indicate proximity to saturation, and sequences with a similarity score of more than 97 percent are assigned to an OTU. MSH: During this treatment, mosquitoes exclusively drank water from the Meishe River. 3HKT and HR3: In this treatment, mosquitoes were kept in water from the Meishe River that had been supplemented with recombinant *Chlamydomonas* 3HKT-3 and HR3-1, respectively. CC48: In this treatment, mosquitoes were kept in water from the Meishe River that had been supplemented with *C. reinhardtii* CC48.

**Additional file 3: Figure S3.** Genus (A, C, E and G) and OTU (B, D, F and H) richness within different groups of MSH, CC48, 3HKT and HR3. MSH: In this treatment, mosquitoes were reared in Meishe River water. CC48: In this treatment, mosquitoes were reared in water supplemented with *C. reinhardtii* CC48. 3HKT and HR3: In this treatment, mosquitoes were raised in water supplemented with recombinant *Chlamydomonas* 3HKT-3 and HR3-1, respectively.

**Additional file 4: Figure S4.** Top 20 genera of microalgae found in test waters from MSH(A), CC48(B), 3HKT(C) and HR3 (D). MSH: In this treatment, mosquitoes were raised in Meishe River water. CC48: In this treatment, mosquitoes were reared in water supplemented with *C. reinhardtii* CC48. 3HKT and HR3: In this treatment, mosquitoes were reared in water supplemented with recombinant *Chlamydomonas* 3HKT-3 and HR3-1, respectively.

**Additional file 5: Table S1.** Water quality detection of Meishe River, Shapo Reservoir, and Hongcheng Lake.

**Additional file 6: Table S2.** The total numbers of assigned phylum, class, genus and OTU in 18S high-throughput sequencing.

## Data Availability

The manuscript and its supporting information files contain all necessary information.
