# Supplementary figures and images for: Effect of marker-free transgenic Chlamydomonas on the control of Aedes mosquito population and on plankton
Source: Parasit Vectors. 2023 Jan 18;16:18. doi: 10.1186/s13071-022-05647-3 (PMC9847121; doi:10.1186/s13071-022-05647-3)

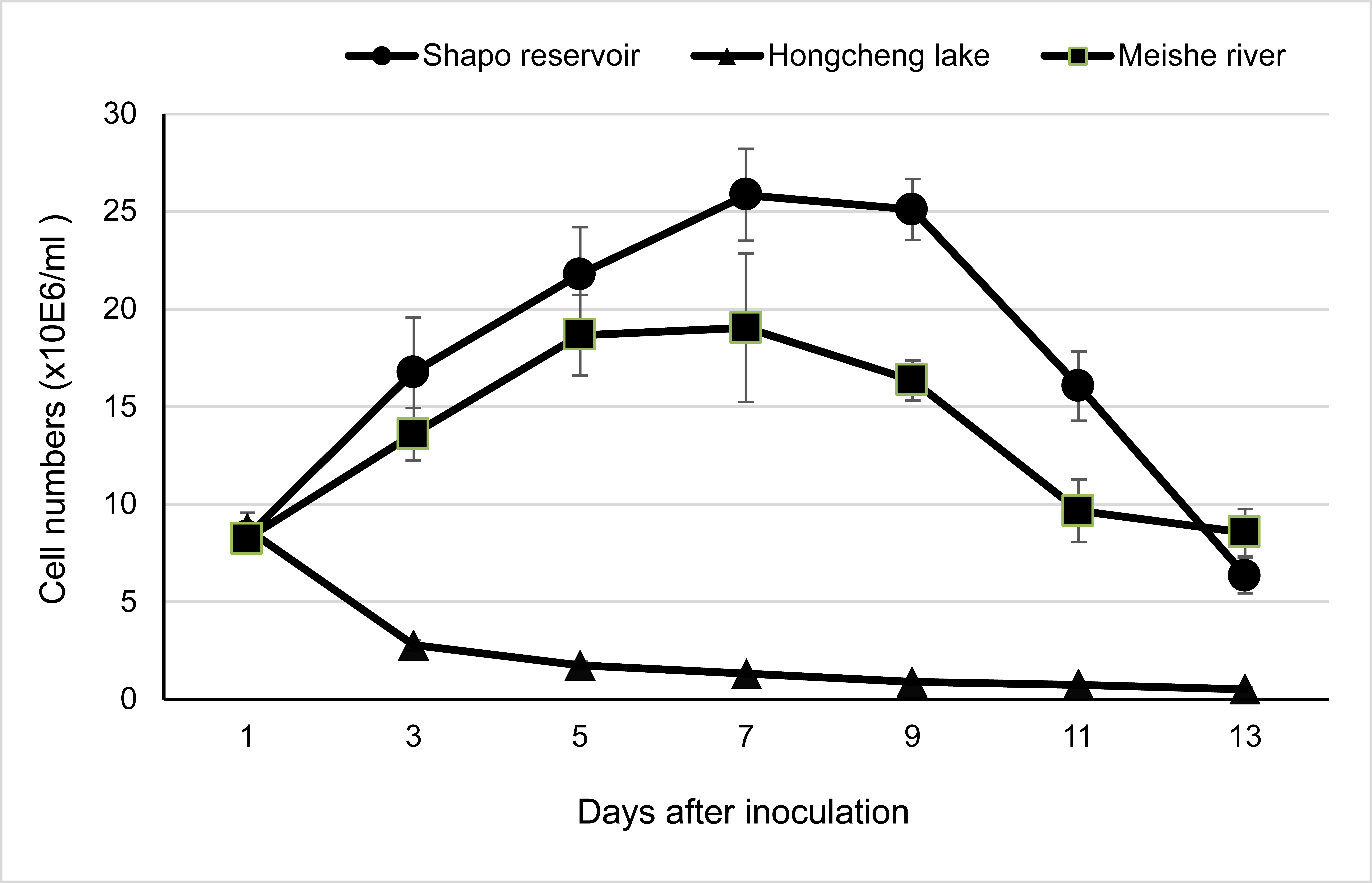

Supplement: Supplementary file 1 — Additional file 1: Figure S1. The growth curve of Chlamydomonas CC48 in the water from Shapo Reservoir, Hongcheng Lake and Meishe River. [file 13071_2022_5647_MOESM1_ESM.tif]

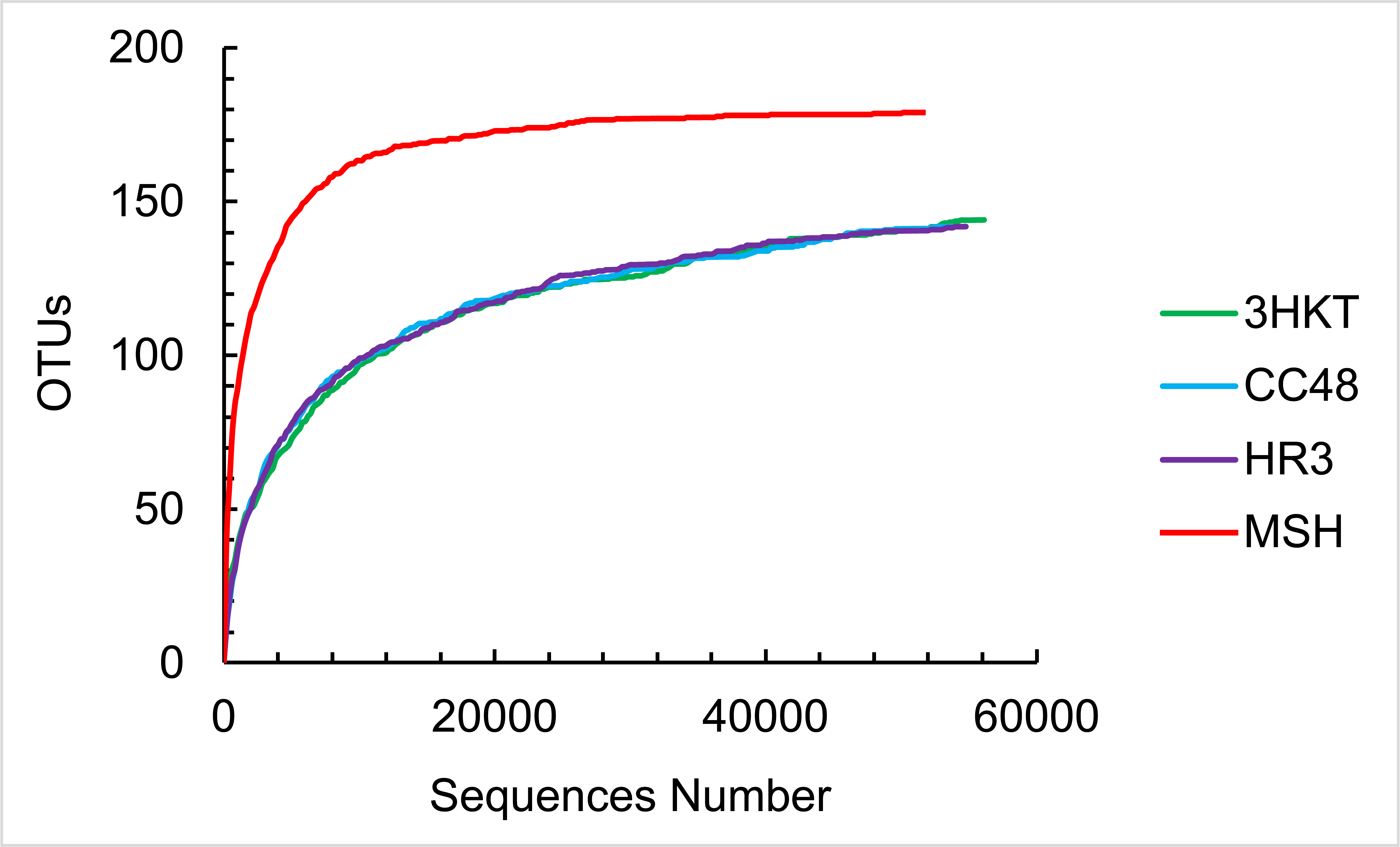

Supplement: Supplementary file 2 — Additional file 2: Figure S2. Rarefaction curves for all samples taken from the test waters' operational taxonomic units (OTUs). Weak slopes at the end of rarefaction curves indicate proximity to saturation, and sequences with a similarity score of more than 97 percent are assigned to an OTU. MSH: During this treatment, mosquitoes exclusively drank water from the Meishe River. 3HKT and HR3: In this treatment, mosquitoes were kept in water from the Meishe River that had been supplemented with recombinant Chlamydomonas 3HKT-3 and HR3-1, respectively. CC48: In this treatment, mosquitoes were kept in water from the Meishe River that had been supplemented with C. reinhardtii CC48. [file 13071_2022_5647_MOESM2_ESM.tif]

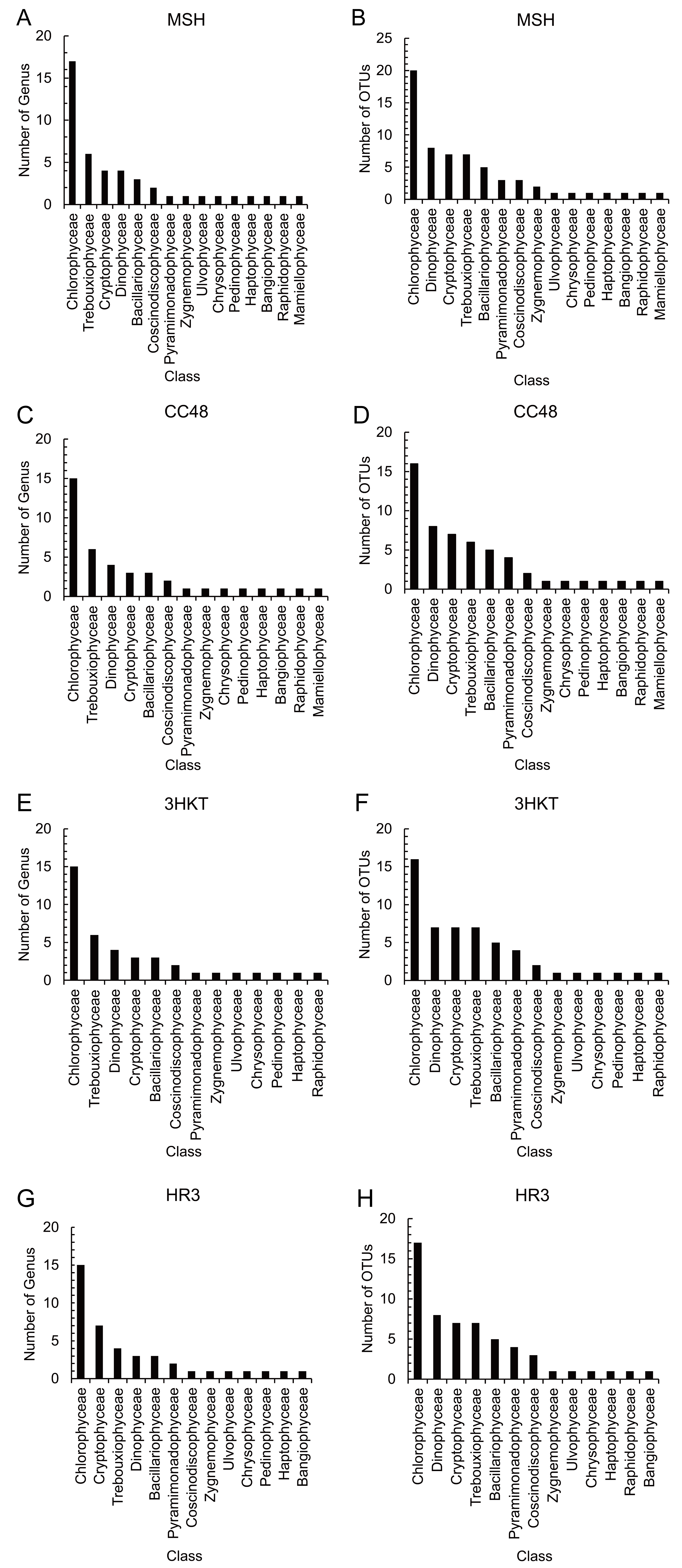

Supplement: Supplementary file 3 — Additional file 3: Figure S3. Genus (A, C, E and G) and OTU (B, D, F and H) richness within different groups of MSH, CC48, 3HKT and HR3. MSH: In this treatment, mosquitoes were reared in Meishe River water. CC48: In this treatment, mosquitoes were reared in water supplemented with C. reinhardtii CC48. 3HKT and HR3: In this treatment, mosquitoes were raised in water supplemented with recombinant Chlamydomonas 3HKT-3 and HR3-1, respectively. [file 13071_2022_5647_MOESM3_ESM.tif]

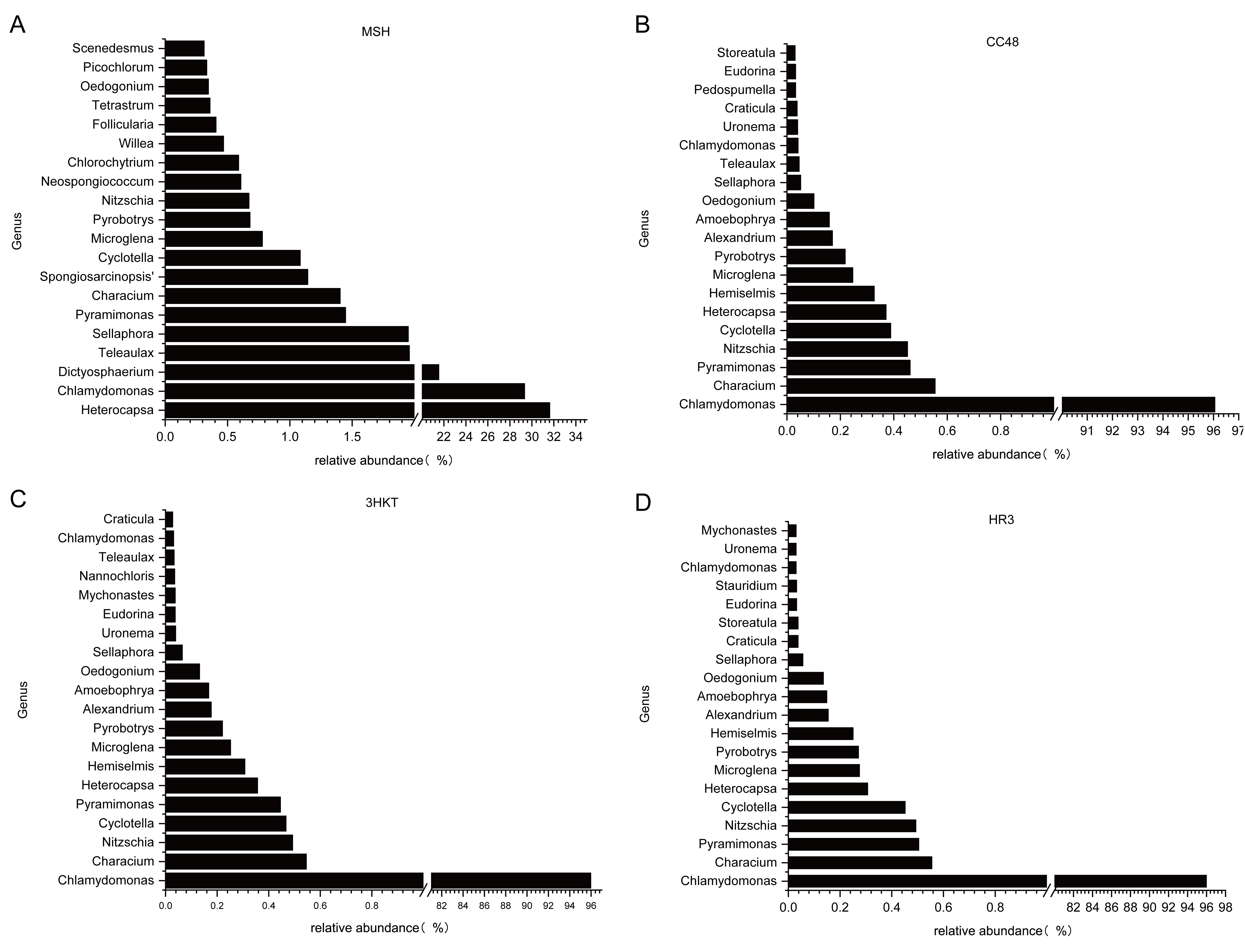

Supplement: Supplementary file 4 — Additional file 4: Figure S4. Top 20 genera of microalgae found in test waters from MSH(A), CC48(B), 3HKT(C) and HR3 (D). MSH: In this treatment, mosquitoes were raised in Meishe River water. CC48: In this treatment, mosquitoes were reared in water supplemented with C. reinhardtii CC48. 3HKT and HR3: In this treatment, mosquitoes were reared in water supplemented with recombinant Chlamydomonas 3HKT-3 and HR3-1, respectively. [file 13071_2022_5647_MOESM4_ESM.tif]

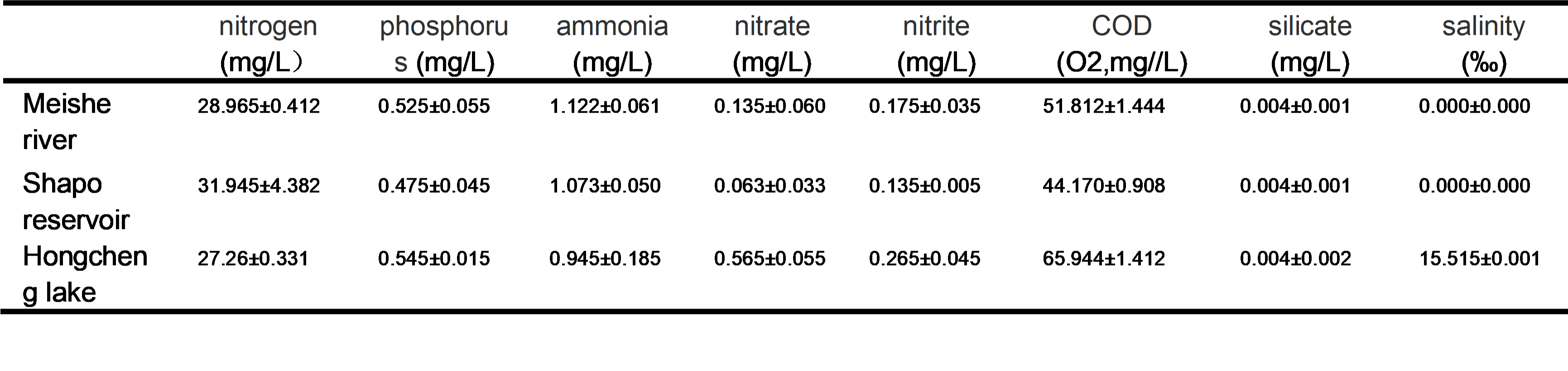

Supplement: Supplementary file 5 — Additional file 5: Table S1. Water quality detection of Meishe River, Shapo Reservoir, and Hongcheng Lake. [file 13071_2022_5647_MOESM5_ESM.tif]

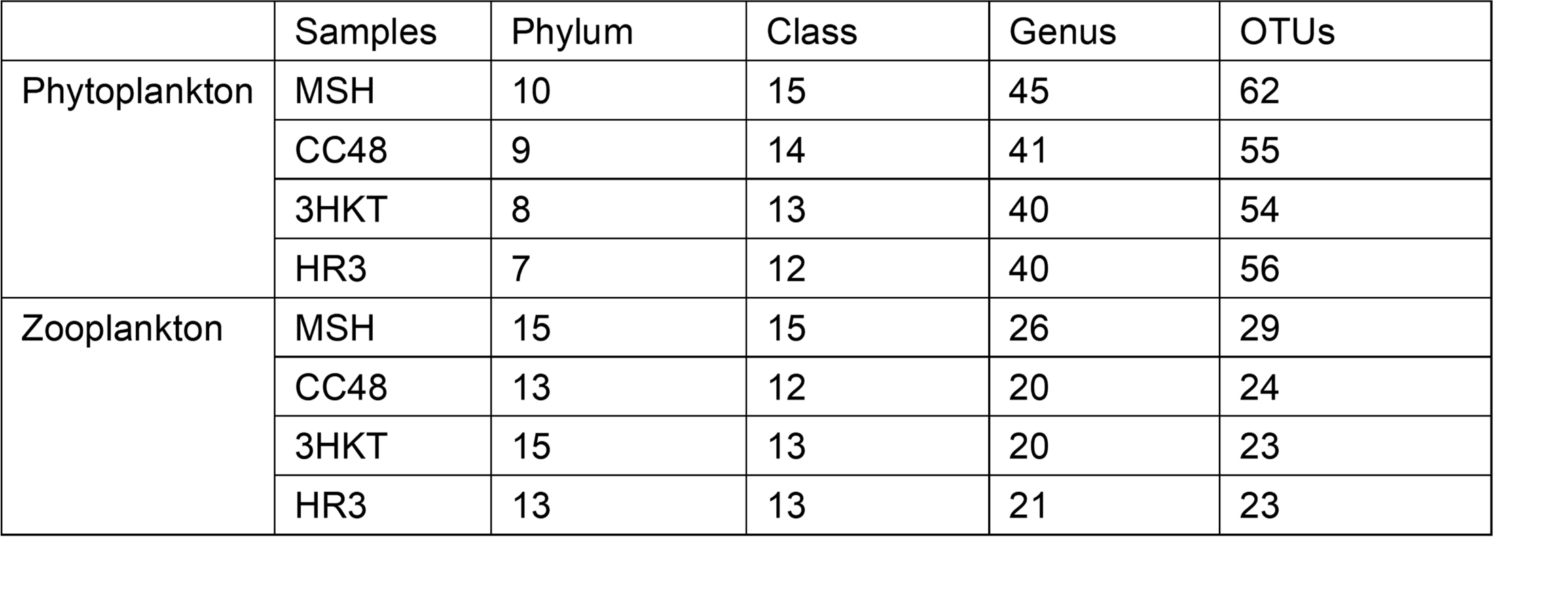

Supplement: Supplementary file 6 — Additional file 6: Table S2. The total numbers of assigned phylum, class, genus and OTU in 18S high-throughput sequencing. [file 13071_2022_5647_MOESM6_ESM.tif]
